# Papillophlebitis Presenting As Optic Disc Edema Following Cervical Manipulation: A Case Report

**DOI:** 10.7759/cureus.102793

**Published:** 2026-02-01

**Authors:** Hisanori Miyashita, Eri Shibuya, Hiroshi Sasaki, Eri Kubo

**Affiliations:** 1 Department of Ophthalmology, Kanazawa Medical University, Ishikawa, JPN; 2 Dapartment of Ophthalmology, Kanazawa Medical University, Ishikawa, JPN

**Keywords:** cervical manipulation, optic disc edema, papillophlebitis, retinal venous congestion, systemic corticosteroid therapy

## Abstract

Papillophlebitis is an uncommon inflammatory disorder of the retinal venous system that typically affects young adults but can also present in older individuals. We report the case of a 68-year-old woman who developed unilateral optic disc edema following cervical manipulation. The best-corrected visual acuity was 20/20 and 20/13 in the right and left eyes, respectively, with no relative afferent pupillary defect. Fluorescein angiography revealed disc leakage without capillary nonperfusion, and optical coherence tomography showed marked peripapillary retinal nerve fiber layer thickening without macular involvement. Brain and orbital magnetic resonance imaging revealed no abnormalities, including intracranial hypertension or venous sinus thrombosis. Based on the clinical and imaging findings, papillophlebitis was diagnosed, and oral corticosteroid therapy was initiated, resulting in the rapid resolution of disc edema and normalization of visual field findings within one month. This case highlights that a clinical picture highly suggestive of papillophlebitis can occur in older adults, potentially triggered by transient circulatory stress, and underscores the effectiveness of corticosteroid therapy in achieving complete anatomical and functional recovery of the optic nerve.

## Introduction

Optic disc edema (ODE) due to venous congestion often raises concerns about serious etiologies such as papilledema, optic neuritis, and central retinal vein occlusion (CRVO). However, some patients exhibit marked disc swelling with preserved visual acuity and no intracranial abnormalities, making clinical differentiation challenging. Papillophlebitis, first described by Lyle and Wybar in 1961 [1], is a localized perivenous inflammatory process at the optic nerve head that leads to transient impairment of venous outflow. Although most frequently reported in young adults, several studies have indicated that similar presentations may occur in older individuals. Hayreh later emphasized the vascular and inflammatory components underlying this condition [2].

The terminology and conceptual framework of papillophlebitis have been re-evaluated, with some authors proposing the term venous papillopathy to better reflect the underlying pathophysiology and to distinguish this entity from incipient central retinal vein occlusion [3]. This distinction is clinically important, particularly in older patients in whom thrombotic or ischemic venous disorders are more prevalent.

Cervical manipulation has been reported to induce transient alterations in vertebrobasilar or ophthalmic circulation [4]. Although a causal relationship between cervical manipulation and retinal venous disorders has not been firmly established, transient changes in venous outflow or venous pressure may theoretically contribute to retinal venous congestion in susceptible individuals. Nevertheless, the association between cervical manipulation and ocular venous pathology remains unclear.

Herein, we report a case of papillophlebitis presenting as optic disc edema in a 68-year-old woman following chiropractic cervical manipulation. This case highlights the importance of recognizing inflammatory venous papillopathy in older adults and carefully distinguishing this entity from papilledema and CRVO, as management strategies and prognostic implications differ substantially.

## Case presentation

A 68-year-old woman with no systemic illness initially presented with unilateral optic disc swelling in her right eye. She had no history of hypertension, diabetes mellitus, atherosclerotic cardiovascular disease, or other systemic vascular risk factors. Six days before presentation, she underwent cervical manipulation at a chiropractic clinic for numbness in her right hand. The following day, she experienced subtle visual distortions and discomfort in her right eye. Two days before the referral, a local ophthalmologist noted ODE in the right eye and immediately referred the patient to our department. Overall, the clinical course followed a clear temporal sequence, with symptom onset one day after cervical manipulation, initial ophthalmic evaluation within one week, initiation of corticosteroid therapy approximately two weeks after onset, and complete anatomical and functional recovery within one month.

**Figure 1:**
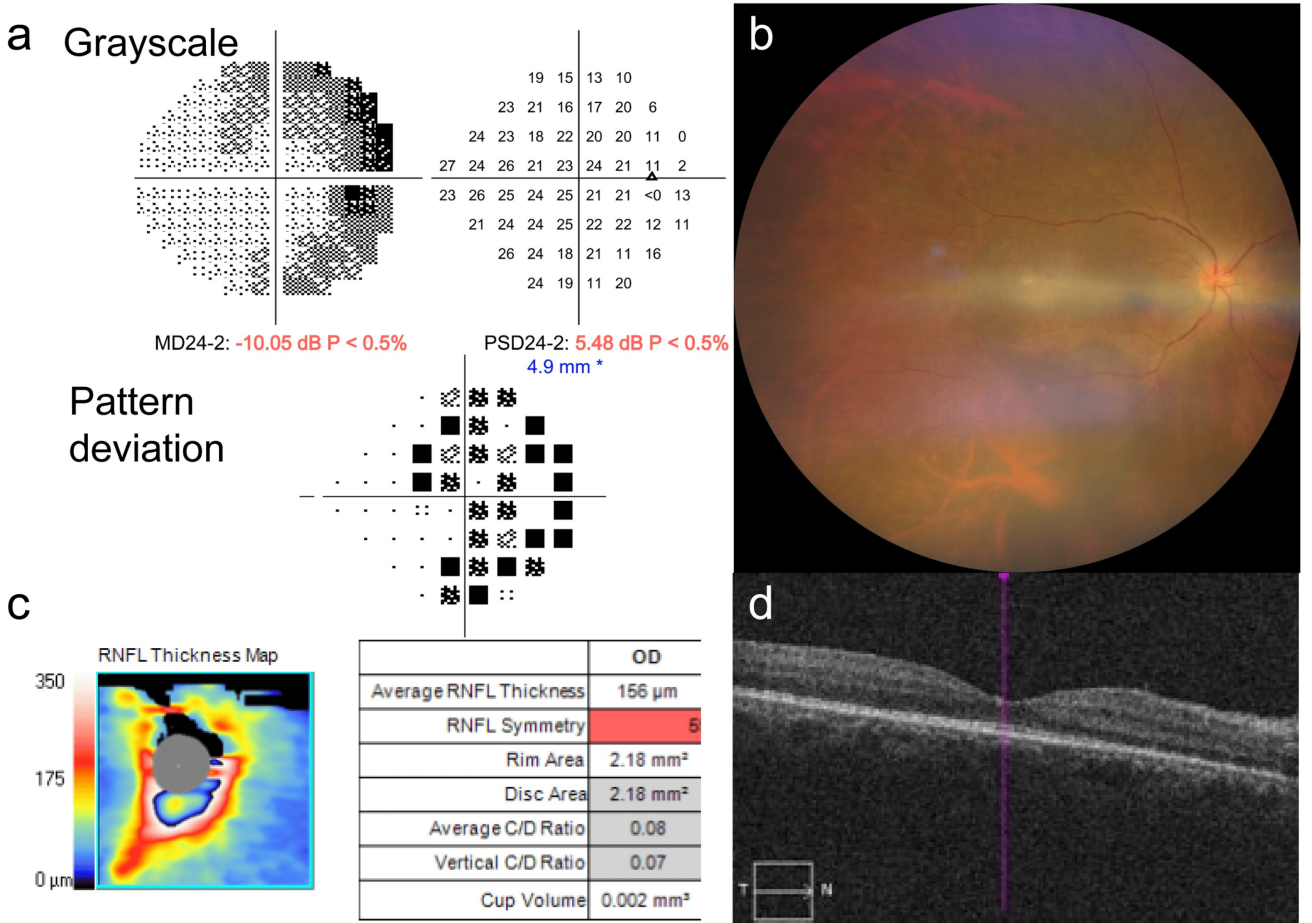
Clinical Imaging Findings at the Initial Visit. (a) Humphrey visual field showing mild enlargement of the physiological blind spot, extending nasosuperiorly. (b) Multicolor fundus image demonstrating marked optic disc edema with venous dilation and tortuosity without retinal hemorrhage. (c) Peripapillary retinal nerve fiber layer (RNFL) thickness map revealing significant RNFL thickening and optic nerve head swelling (average RNFL thickness: 156 µm). (d) SD-OCT B-scan showing preserved macular architecture without macular involvement despite the presence of optic disc edema. SD-OCT: Spectral-domain optical coherence tomography

At presentation, the best-corrected visual acuity (BCVA) was 20/20 and 20/13 in the right and left eyes, respectively. There was no pain with ocular movements or any relative afferent pupillary defect. The central flicker fusion frequency was within the normal range for both eyes. Humphrey 24-2 visual field testing was performed using an automated perimeter (Carl Zeiss Meditec, Dublin, CA, USA) and revealed mild enlargement of the physiological blind spot extending nasosuperiorly in the right eye, whereas the left visual field was unremarkable (Figure 1a).

Fundus examination and multicolor fundus imaging (ZEISS CLARUS 700, Carl Zeiss Meditec) of the right eye revealed severe inflammation of the optic nerve head with retinal venous vasodilatation and tortuosity but no hemorrhage in any quadrant (Figure 1b). Fluorescein angiography (FA) showed late dye leakage from the optic disc, indicating increased disc permeability without evidence of venous occlusion, capillary nonperfusion, or ischemic retinal changes. Spectral-domain optical coherence tomography (SD-OCT; ZEISS CIRRUS 5000, Carl Zeiss Meditec) confirmed marked papillary edema without macular involvement (Figure 1c, 1d).

The intraocular pressure, measured using a non-contact tonometer, was 13 mmHg in both eyes. Laboratory testing, including angiotensin-converting enzyme (ACE), anti-neutrophil cytoplasmic antibody (ANCA), anti-nuclear antibody (ANA), complete blood count (CBC), syphilis serology, and C-reactive protein (CRP), was unremarkable, except for an anti-aquaporin-4 (AQP4) antibody level of 3.8 units/L (upper limit of normal: 3.0 units/L). The mildly elevated anti-AQP4 immunoglobulin G level was considered clinically insignificant, as the patient lacked clinical or radiological features suggestive of neuromyelitis optica spectrum disorder, and the finding was interpreted as a possible low-level false-positive result. The laboratory findings are summarized in Table 1.

**Table 1:** Laboratory findings at the initial visit Summary of laboratory test results at the initial visit, including inflammatory markers, autoimmune antibodies, and infectious screening, with corresponding normal reference ranges. The results revealed no laboratory evidence of systemic inflammation or autoimmune disease relevant to the differential diagnosis of optic disc edema. ACE: Angiotensin-Converting Enzyme; CRP: C-Reactive Protein; AQP4-IgG: Aquaporin-4 Immunoglobulin G; ANCA: Anti-Neutrophil Cytoplasmic Antibody; ANA: Anti-Nuclear Antibody; CBC: Complete Blood Count.

Test	Result	Normal range
ACE	7.4 U/L	8.3–21.4 U/L
CRP	<0.02 mg/dL	0.00–0.14 mg/dL
AQP4-IgG	3.8 U/L	<3.0 U/L
ANCA	Negative	Negative
ANA	Negative	Negative
Syphilis serology	Negative	Negative
CBC	Within normal limits	

Five days later, brain and orbital magnetic resonance imaging revealed no abnormalities, including no evidence of mass lesions, demyelination, venous sinus thrombosis, or signs of raised intracranial pressure, such as empty sella or posterior globe flattening. Differential diagnoses, including non-arteritic anterior ischemic optic neuropathy, optic neuritis, papilledema, and incipient central retinal vein occlusion, were considered and excluded based on preserved visual acuity, absence of pain with eye movement, unilateral involvement, characteristic imaging findings, and normal neuroimaging. Although the erythrocyte sedimentation rate was not measured, giant cell arteritis was considered unlikely, given the absence of systemic symptoms and normal inflammatory markers. Visual acuity, color vision, and pupillary reflexes remained stable; however, subsequent fundus examination showed progressive ODE with irregular venous caliber and increased vascular tortuosity in the right eye.

**Figure 2:**
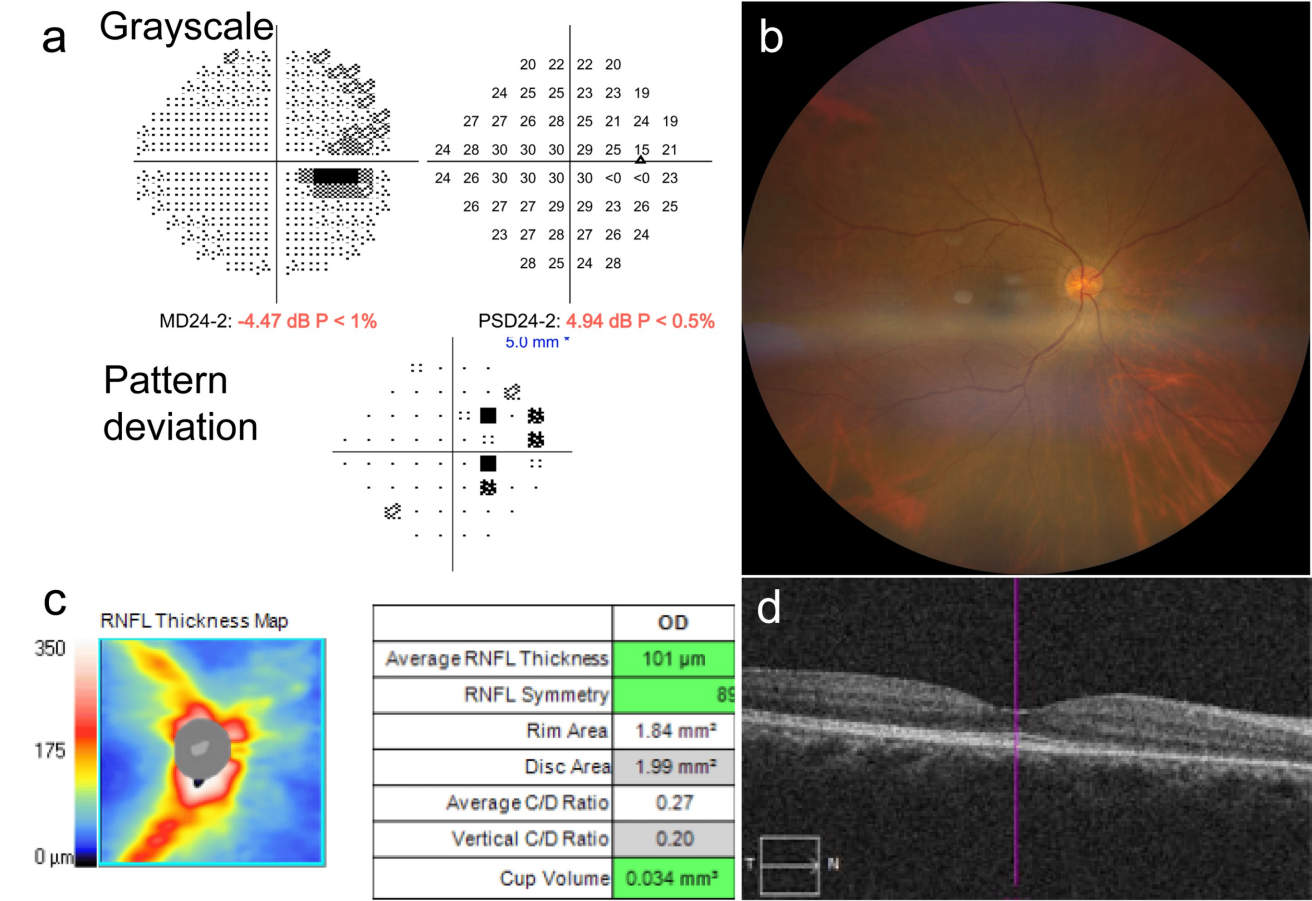
Clinical Imaging Findings on day 42 of follow-up. (a) Humphrey visual field revealing resolution of the previously enlarged blind spot and restoration of normal visual-field sensitivity. (b) Multicolor fundus image showing marked resolution of optic disc edema following systemic corticosteroid therapy. (c) RNFL thickness map demonstrating a reduction in RNFL swelling, with an average thickness of 101 µm. (d) SD-OCT B-scan indicating near-complete normalization of the optic disc morphology. RNFL: Retinal nerve fiber layer; SD-OCT: Spectral-domain optical coherence tomography

Based on these findings, a diagnosis of venous papillopathy strongly suggestive of inflammatory papillophlebitis was considered, and oral prednisolone (40 mg/day) was initiated. After 12 days of treatment, BCVA improved to 20/13 in both eyes, and optic disc swelling was markedly reduced on follow-up fundus examination and SD-OCT imaging. The prednisolone dose was tapered to 35 mg/day. By day 19, further resolution of disc edema was observed, and the dose was gradually reduced from 30 mg/day to 25 mg/day and then to 20 mg/day. At the one-month follow-up, the BCVA remained 20/13 in both eyes, disc swelling continued to regress, the visual field defect resolved, and SD-OCT demonstrated complete normalization of the optic disc morphology (Figure 2a, 2b, 2c, and 2d).

## Discussion

This case report describes an unusual presentation of steroid-responsive optic disc edema with clinical features consistent with papillophlebitis in an older woman following cervical manipulation. These findings align with the previously documented characteristics of papillophlebitis, including localized venous inflammation, venous dilation without retinal hemorrhage, preserved visual acuity, and a favorable response to corticosteroid therapy [5-8]. The absence of intracranial abnormalities and maintained visual acuity further distinguished this presentation from papilledema and typical central retinal vein occlusion (CRVO).

Papillophlebitis is thought to result from low-grade inflammation of the retinal venules around the optic disc, leading to transient venous outflow impairment and optic disc edema [2,3,5]. Although this entity has classically been described in young adults, inflammatory venous papillopathies have also been reported across a wider age range, and the concept has been re-evaluated as venous papillopathy to better distinguish it from incipient CRVO [3].

In the present case, CRVO was carefully considered in the differential diagnosis given the patient’s age; however, the absence of systemic vascular risk factors, lack of retinal hemorrhage or ischemic changes on fluorescein angiography, and preserved visual acuity favored an inflammatory venous process rather than a thrombotic event [7]. In addition, fundus examination revealed venous dilation and disc leakage without retinal hemorrhage or venous sheathing, further supporting an inflammatory rather than purely thrombotic mechanism. Comparable inflammatory optic disc conditions with venous congestion have been reported in association with systemic inflammatory diseases [9].

Cervical manipulation has been reported to induce transient alterations in craniocervical circulation. Cervical manipulative therapy has been associated primarily with arterial complications, such as cervical arterial dissection, as emphasized in a scientific statement from the American Heart Association/American Stroke Association [10]. Although these reports concern arterial pathology, they support the concept that cervical manipulation can influence craniocervical vascular dynamics. Such transient hemodynamic changes may theoretically contribute to venous congestion in susceptible individuals. However, a direct causal relationship between cervical manipulation and retinal venous disease cannot be established, and the present association should be interpreted as temporal rather than definitive.

Diagnostic imaging plays a crucial role in differentiating inflammatory venous papillopathy from papilledema, pseudoedema, and ischemic optic neuropathy [11]. In this case, multimodal imaging, including fluorescein angiography and spectral-domain optical coherence tomography, demonstrated disc leakage and RNFL thickening without evidence of venous occlusion or macular involvement, supporting this diagnosis.

To our knowledge, this is the first reported case of papillophlebitis presenting as optic disc edema following cervical manipulation. The rapid improvement following corticosteroid therapy is consistent with an underlying inflammatory mechanism [6,8]; however, treatment response alone should be interpreted as supportive rather than definitive evidence of diagnosis. Overall, this case expands the clinical spectrum of papillophlebitis-like presentations to include older adults and underscores the importance of careful differential diagnosis in cases of optic disc edema with preserved visual function.

## Conclusions

This case highlights that a clinical presentation highly suggestive of papillophlebitis may occur in older adults and may be precipitated by transient circulatory or mechanical stress, such as cervical manipulation. Accurate differentiation from CRVO, ischemic optic neuropathy, and papilledema is essential, particularly when visual acuity is preserved. Multimodal imaging, including SD-OCT and FA, is critical for the evaluation and follow-up of these patients. Although the absence of cerebrospinal fluid pressure measurements and hemodynamic imaging prevents the complete exclusion of all secondary causes, the rapid and robust response to corticosteroid therapy supports an underlying inflammatory venous mechanism of the disease. This case broadens the recognized clinical spectrum of papillophlebitis-like presentations in older patients and underscores the potential effectiveness of prompt corticosteroid treatment.
